# Renal tubular gen e biomarkers identification based on immune infiltrates in focal segmental glomerulosclerosis

**DOI:** 10.1080/0886022X.2022.2081579

**Published:** 2022-06-17

**Authors:** JunYuan Bai, XiaoWei Pu, YunXia Zhang, Enlai Dai

**Affiliations:** a Medical College of Integrated Chinese and Western Medicine, GanSu University of Traditional Chinese Medicine, GanSu, China; b Department of Anesthesiology and Surgery, GanSu University of Traditional Chinese Medicine, Gansu, China

**Keywords:** Focal segmental glomerulosclerosis, tubular, immune infiltrates, machine learning, nuclear receptor subfamily 4 group A member 1, dual specificity phosphatase 1

## Abstract

**Objective:**

The present study identified novel renal tubular biomarkers that may influence the diagnosis and treatment of focal segmental glomerulosclerosis (FSGS) based on immune infiltration.

**Methods:**

Three FSGS microarray datasets, GSE108112, GSE133288 and GSE121211, were downloaded from the Gene Expression Omnibus (GEO) database. The R statistical software limma package and the combat function of the sva package were applied for preprocessing and to remove the batch effects. Differentially expressed genes (DEGs) between 120 FSGS and 15 control samples were identified with the limma package. Disease Ontology (DO) pathway enrichment analysis was conducted with statistical R software to search for related diseases. Gene set enrichment analysis (GSEA) was used to interpret the gene expression data and it revealed many common biological pathways. A protein-protein interaction (PPI) network was built using the Search Tool for the Retrieval of Interacting Genes (STRING) database, and hub genes were identified by the Cytoscape (version 3.7.2) plug-in CytoHubba. The plug-in Molecular Complex Detection (MCODE) was used to screen hub modules of the PPI network in Cytoscape, while functional analysis of the hub genes and hub nodes involved in the submodule was performed by ClusterProfiler. The least absolute shrinkage and selection operator (LASSO) regression and support vector machine recursive feature elimination (SVM-RFE) analysis were used to screen characteristic genes and build a logistic regression model. Receiver operating characteristic (ROC) curve analyses were used to investigate the logistic regression model and it was then validated by an external dataset GSE125779, which contained 8 FSGS samples and 8 healthy subjects. Cell-type identification by estimating relative subsets of RNA transcripts (CIBERSORT) was used to calculate the immune infiltration of FSGS samples.

**Results:**

We acquired 179 DEGs, 79 genes with downregulated expression (44.1%) and 100 genes with upregulated expression (55.9%), in the FSGS samples. The DEGs were significantly associated with arteriosclerosis, kidney disease and arteriosclerotic cardiovascular disease. GSEA revealed that these gene sets were significantly enriched in allograft rejection signaling pathways and activation of immune response in biological processes. Fifteen genes were demonstrated to be hub genes by PPI, and three submodules were screened by MCODE linked with FSGS. Analysis by machine learning methodologies identified nuclear receptor subfamily 4 group A member 1 (NR4A1) and dual specificity phosphatase 1 (DUSP1) as sensitive tubular renal biomarkers in the diagnosis of FSGS, and they were selected as hub genes, as well as hub nodes which were enriched in the MAPK signaling pathway. Immune cell infiltration analysis revealed that the genetic biomarkers were both correlated with activated mast cells, which may amplify FSGS biological processes.

**Conclusion:**

DUSP1 and NR4A1 were identified as sensitive potential biomarkers in the diagnosis of FSGS. Activated mast cells have a decisive effect on the occurrence and development of FSGS through tubular lesions and tubulointerstitial inflammation, and they are expected to become therapeutic targets in FSGS.

## 1. Introduction

Focal segmental glomerulosclerosis is a common regular renal disease that can lead to steroid-resistant nephrotic syndrome (SRNS) in both adults and children [1]. The incidence of FSGS varies from 1.4 to 21 cases per million people and it can occur in any age group; approximately 7–10% of children and 20–30% of adults have nephrotic syndrome [2]. Untreated or primary FSGS often presents with progressive renal inadequacy and progresses to end-stage renal disease (ESRD). FSGS causes considerable clinical and economic burdens, and the presence of proteinuria in the field of nephropathy would also increase the economic burden [3].

The majority of patients with FSGS suffer from nonselective proteinuria, hypertension, renal impairment and renal tubular dysfunction. Pathologically, patients with FSGS often present with severe tubulointerstitial lesions. Previous research has linked the degree of tubulointerstitial injury with glomerular injury and subsequent renal scarring formation [4]. Previous studies have shown that an increased rate of tubular apoptosis in a kidney primary biopsy is an independent predictor of early FSGS progression to ESRD [5]. Chronic tubulointerstitial lesions affect the FSGS prognosis, and tubular interstitial fibrosis is an independent risk factor for decreased renal function in patients with FSGS.

Current therapeutic molecular pathways include inhibitors of the renin-angiotensin-aldosterone axis (RAAS), sodium-glucose cotransporter 2 (SGLT2), endothelin (ET), and novel pathways such as tumor necrosis factor (TNF), Janus kinase/signal transducer and activator of transcription (JAK-STAT) signaling [6]. The existence of cyclic permeability in the plasma of FSGS patients suggests that autoantibody reactivity is the main cause of primary FSGS. During nephrotic syndrome, the leakage of plasma protein into the urinary cavity leads to immediate local tissue damage, such as increased extracellular matrix and the formation of interstitial fibrosis [7]. Studies have indicated that infiltrating immune cells, including antibodies against T cells, B cells and macrophages, were found in renal biopsies from NS [8], while the deposition of complement C3 was found in the proximal tubules of FSGS [9].

Proteinuria is a significant driving factor for the progression of tubulointerstitial inflammation and fibrosis, leading to the activation of proximal tubular inflammatory responses [10], which are performed by several intracellular signaling pathways, such as induction of tubular chemokine expression, tubular epithelial cell atrophy/apoptosis induced by endoplasmic reticulum stress, oxidative stress, inflammatory cell filtration in the interstitium and persistent fibrosis [11]. Previous studies have identified biomarkers in the proximal tubules, such as megalin, cubilin, the neonatal Fc receptor (FcRn), CD36, CD44, neutrophil gelatinase-associated lipocalin (NGAL), kidney injury molecule-1 (KIM-1), fatty acid transporter-2 (FATP2). Megalin is expressed on the apical membrane of proximal tubules and has a fundamental effect on the reabsorption of proteins of various molecular dimensions [12]. Cubillin is essential for tubular absorption of albumin, and megalin is required for the endocytosis of the cubilin-albumin complex [13]. Meanwhile, megalin/cubilin participates in albumin-induced tubular lesions followed by tubulointerstitial inflammation [11]. FcRn has been implicated as the "receptor" mediating albumin transcytosis, in collaboration with megalin and cubilin, primarily selecting proteins for lysosomal degradation [14]. CD36 is expressed in tubular epithelial cells and it affects kidney lipid metabolism as well as the binding and uptake of albumin in the proximal tubule, is significantly upregulated in chronic kidney disease (CKD), and it plays a significant role both in the diagnosis and therapy of renal fibrosis [15,16]. Albumin induces proximal tubular epithelial cells (PECs) to express CD44 by activating the ERK signaling pathway [17]. The increase in NGAL production and release from tubular cells after harmful stimuli of various kinds, which levels clearly correlate with the severity of renal impairment, probably expressing the degree of active damage underlying the chronic condition [18]. KIM-1 is a scavenger receptor that is upregulated on the apical membrane of proximal tubules in proteinuric kidney disease [19]. NGAL is a biomarker of distal tubular segments, while KIM-1 is a biomarker that originated from proximal tubules [20]. FATP2 is an important apical proximal tubule nonesterified fatty acid transporter that regulates lipoapoptosis and it may be a target for the prevention of CKD progression [21]. Researchers have confirmed [22] that urine is a valuable source of proteins and metabolites, in which a decrease in α-1 antitrypsin, E-cadherin, 39S ribosomal protein L17, histatin-3 and matrix-remodeling protein 8 and an increase in transferrin, uromodulin, calretinin ubiquitin-60S ribosomal protein L40 and apolipoprotein-A1 can be considered potential biomarkers of FSGS.

Traditional studies are mostly based on a single gene detection mechanism, and there is a lack of studies evaluating multiple genes and pathways during the formation of FSGS. To enable a better understanding of the immune mechanisms involved in tubulointerstitial fibrosis and to investigate the pathogenesis and mechanism of FSGS, in this study, machine learning methodologies were applied to perform complete bioinformatics analysis.

## 2. Materials and methods

We obtained relevant gene chips from the GEO database (https://www.ncbi.nlm.nih.gov/geo/), which are freely available. The DEGs were analyzed using the limma package with standard data processing. DO analysis was carried out by statistical R-software to search for related diseases associated with FSGS. GSEA was applied to reveal the gene sets and biological pathways enriched in FSGS. The PPI network was then executed on the STRING website (https://cn.string-db.org/). Cytoscape software was used to identify hub genes. MCODE was used to screen submodules of the PPI network. Meanwhile, Gene Ontology (GO) functional annotation of the hub genes and Kyoto Encyclopedia of Genes and Genomes (KEGG) signaling pathway enrichment analysis of the hub nodes involved in the submodule was performed by ClusterProfiler. LASSO regression and SVM-RFE analysis were used to screen for characteristic genes and build a logistic regression model to make the results more accurate and standardized. ROC curve analysis was used to investigate the logistic regression model, which was validated by an external dataset. Then, the CIBERSORT algorithm was applied to calculate the immune infiltration of the FSGS samples.

### 2.1. Acquisition of gene expression data files

The FSGS series of GSE108112, GSE133288 and GSE121211 were downloaded from the GEO database and they contain tubulointerstitial transcriptome expression profiles. GSE108112 was annotated by GPL19983 as a Series Matrix File, including 46 FSGS and 5 control samples. The same as GSE108112, GSE133288 was also annotated by GPL19983, including 69 FSGS patients and 5 control samples. The GSE121211 Series Matrix File was annotated by GPL17586, involving 5 FSGS patients and 5 control samples. Global analysis of gene expression patterns in datasets of GSE133288 and GSE108112 was performed by Affymetrix Human Gene 2.1 ST Array, while datasets of GSE121211 were obtained from the Affymetrix HTA 2.0 microarray. The R statistical software limma package [23] and the combat function of the sva package [24] were applied to preprocess and remove the batch effects of these three datasets. After integrating the three profile datasets, we identified a total of 20178 expressed genes.

### 2.2. Identification of DEGs associated with FSGS

In the present study, to determine the DEGs between FSGS and healthy tissues, the adj. *p* < 0.05 and |logFold Change| > 1 were selected as the cutoff criteria. The R statistical software “limma” package was applied to extract the DEGs from genes we identified in the integrated dataset, while the “pheatmap” and “ggplot2” packages [25] were applied to construct the heatmap and volcano plot to visualize these DEGs.

### 2.3. DEGs disease ontology pathway enrichment analysis in FSGS

DO pathway enrichment analysis was conducted with R statistical software “clusterProfiler” [26], “org.Hs.eg.db,” “DOSE” [27] and “enrichplot” packages to analyze the DEGs and discover disease associations of the integrated dataset. Then, the “ggplot2” package was applied to create a barplot to visualize the core enriched diseases.

### 2.4. DEGs gene set enrichment analysis in FSGS

To interpret the gene expression data and reveal the many biological pathways in common by focusing on gene sets, DEG-related GO and KEGG enrichment analyses were performed by GSEA [28], and comprehensive bioinformatics analyses were conducted *via* the “limma,” “clusterProfiler” and “org.Hs.eg.db” packages of R. The “c2.cp.kegg.v7.4.symbols.gmt” and “c5.go.v7.4.symbols.gmt” were downloaded from the Molecular Signatures Database (MSigDB) and used as background gene set data.

### 2.5. PPI network construction and submodule analysis

The online database STRING [29] was applied to construct a PPI network of the DEGs. In addition to exploring the relationships among the DEGs, a confidence score >0.7 was set as significant. Cytoscape software [30] was then employed to analyze the interactive relationships of the candidate proteins and visualize the PPI network. A novel Cytoscape plugin cytoHubba [31] ranked nodes in a network by their network features, and the maximal clique centrality (MCC) algorithm was applied to identify the hub genes in this study.

Plug-in MCODE [32] was utilized to identify the hub modules of the PPI network, with the criteria of degree cutoff = 2, node score cutoff = 0.2, k-core = 2, max depth = 100 and the minimum number of genes ≥4 to recognize the main clustering modules as well as the most prominent clustering modules. Then, functional analysis of the hub nodes involved in the submodule was performed by ClusterProfiler.

### 2.6. Construction of the LASSO model and SVM-RFE of candidate gene biomarkers in FSGS

Based on the DEGs, the present study utilized two machine learning algorithms to screen characteristic genes and construct diagnostic classifiers to mine the genes associated with FSGS. LASSO regression [33] uses regularization to improve the prediction accuracy and was carried out with the “glmnet” package of R, for which we set the response type as “binomal” and alpha as “1.” SVM-RFE analysis [34] is a powerful tool for analyzing data with varieties calculators roughly equal to or greater than the number of observations, especially in the RFE-pseudo samples, which can be carried out accurately for the analysis of biomedical data. The SVM classifier was performed using the “e1071,” “kernlab” and “caret” packages of R. Duplicated genes were identified from the two machine learning algorithms, representing candidate gene biomarkers in the renal tubules of FSGS.

### 2.7. Value of gene biomarkers in FSGS

ROC curve analyses [35] were used to investigate the logistic regression model and finally validated by an external dataset GSE125779, which contained 8 FSGS samples and 8 healthy subjects and was annotated by GPL17586. The area under the ROC curve (AUC) synthesized the predictive capacities of each variable, which was applied to evaluate the gene biomarker effectiveness in that subset range to discriminate FSGS from control samples.

### 2.8. Analysis of immune cell infiltration in FSGS

CIBERSORT is a calculation method for the quantification of the cell composition of complex tissues from their gene expression profiles and should enable large-scale analysis of RNA mixtures for cellular biomarkers and therapeutic targets [36]. In the present verification, the immune infiltration of FSGS tissues was calculated by CIBERSORT. The “CIBERSORT” package of R was used to quantify the relative proportion of 22 infiltrating immune cells. Meanwhile, the “corrplot” package of R was applied to conduct the correlation analysis of the immune correlation between the gene biomarkers and immune cells, while the “vioplot” software package was applied to visualize the differences between FSGS patients and controls.

### 2.9. Analysis of the correlation between DEGs and immune cell infiltration

The relationship between the genetic biomarkers we identified and immune cell infiltration was examined by Pearson correlation analysis. The “ggplot2” software package of R was used to visualize the results of the correlation analysis.

## 3. Statistical analysis

According to the data type and characteristics, comparisons were made using the Mann–Whitney *U* test for categorical variables and Student’s *t*-test for continuous variables. R (version 4.1.1) was used to perform all statistical analyses. All statistical analyses accepted *p* < 0.05 for two-sided tests as statistically significant.

## 4. Results

### 4.1. Acquisition of gene expression data files

**Figure 1.**
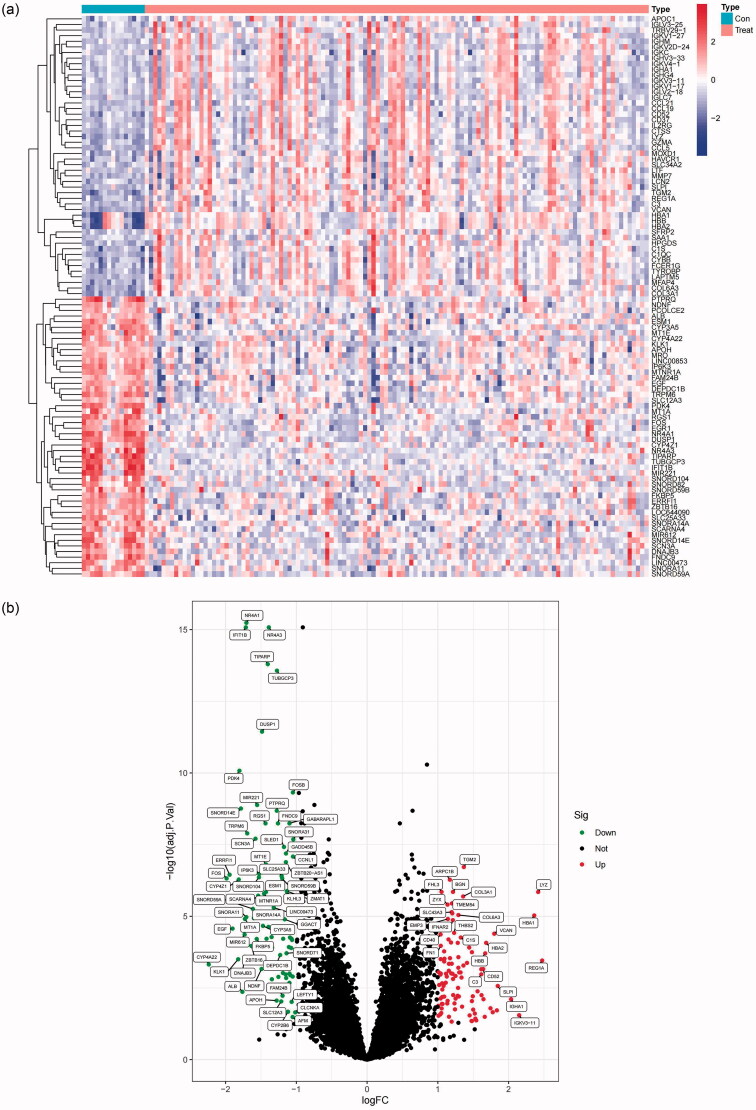
DEGs in tubular cells between FSGS and normal controls. (a) The heatmap shows significant DEGs between FSGS and normal controls. The X-axis represents the sample type, and the Y-axis represents the DEGs. (b) Volcano plot exhibiting DEGs between FSGS and normal controls, downregulated genes and upregulated genes. The X-axis represents the logFC, and the Y-axis represents the -log10 (adj. P.Val). *P* values < 0.05 indicate statistical significance. DEGs: differentially expressed genes.

According to the results of a comprehensive bioinformatics analysis, we acquired 179 DEGs, which included 79 downregulated (44.1%) and 100 upregulated genes (55.9%) in tubulointerstitial tissues from FSGS patients compared to control samples (Table 1). Moreover, these DEGs were visualized by a heatmap and volcano plot (Figure 1).

**Table 1.** The differentially expressed genes in tubular cells between FSGS and normal controls.

DEGS	Genes name
Down-regulated	CYP4A22, FOS, ERRFI1, EGF, KLK1, CYP4Z1, PDK4, ALB, SNORD14E, MIR612, MT1A, DNAJB3, IFIT1B, SNORA11,NR4A1, TRPM6, ZBTB16, SNORD59A, SNORA14A, SCN3A,FKBP5, MIR221, SCARNA4, MRO, SNORD104, IP6K3, NDNF,DUSP1, CYP3A5, MTNR1A, RGS1, MT1E, ESM1, SNORD82,TIPARP, LOC644090, NR4A3, EGR1, PCOLCE2, LINC00853,LINC00473, APOH, PTPRQ, TUBGCP3, FNDC9, DEPDC1B, SLC12A3, SLC25A33, SNORD59B ,FAM24B,TMEM207, RGS2, SLED1, GGACT, SORCS1, ZBTB20-AS1,GADD45B, HRG, SNORD71, ZMAT1, KLHL3, CYP2B6,NUGGC, CYP27B1, GABARAPL1, SNORA79, PTPRO, AFM,SNORD67, N4BP2L2-IT2, CHI3L1, DPY19L2, LEFTY1, CEL, FOSB, SNORA81,CCNL1, SNORA31, CLCNKA
Up-regulated	CAPN6, CST6, LRRN4, APOBEC3G, CPA3, MPEG1, RNASE6,CD40, PLP2, LCP2, FN1, FLT3, FHL3, CD2, CD48, LCP1, C2, OAS3, CD53, CD180, SPIN2B, IFITM1, SLAMF7, APOBEC3F,UCP2, FXYD5, TLR7, IFI6, ZYX, WFDC2, EMP3, LGALS1,HLA-DQA1, C1R, IGLV4-69, ARPC1B, PXDN, TIMP1, TNC,TOP2A, IGHV3-15, SLC43A3, TMEM54, GPX8, PLTP, BGN,IFNAR2, HSPB6, CCL11, THBS2, SFRP2, IGLV3-25, CD37, MFAP4, HPGDS,TRBV29-1, HAVCR1, FCER1G, TYROBP,COL6A3, IL2RG, C1S, LAPTM5, GZMA, CTSS, APOC1,CYBB,COL3A1,TGM2,CCL5,C1QC,IGLV2-18, IGHV3-33,SLC34A2,IGKC, MOXD1, LCN2, LTF, IGHG4, SAA1, IGKV1-27, CCL21,C3, MMP7, CCL19, CD52, IGLC7, IGKV1-17, HBB, HBA2,IGHM, IGKV2D-24, VCAN, IGKV4-1, SLPI, IGHA1, IGKV3-11, HBA1, LYZ, REG1A

### 4.2. DEG disease ontology pathway enrichment analysis in FSGS

**Figure 2.**
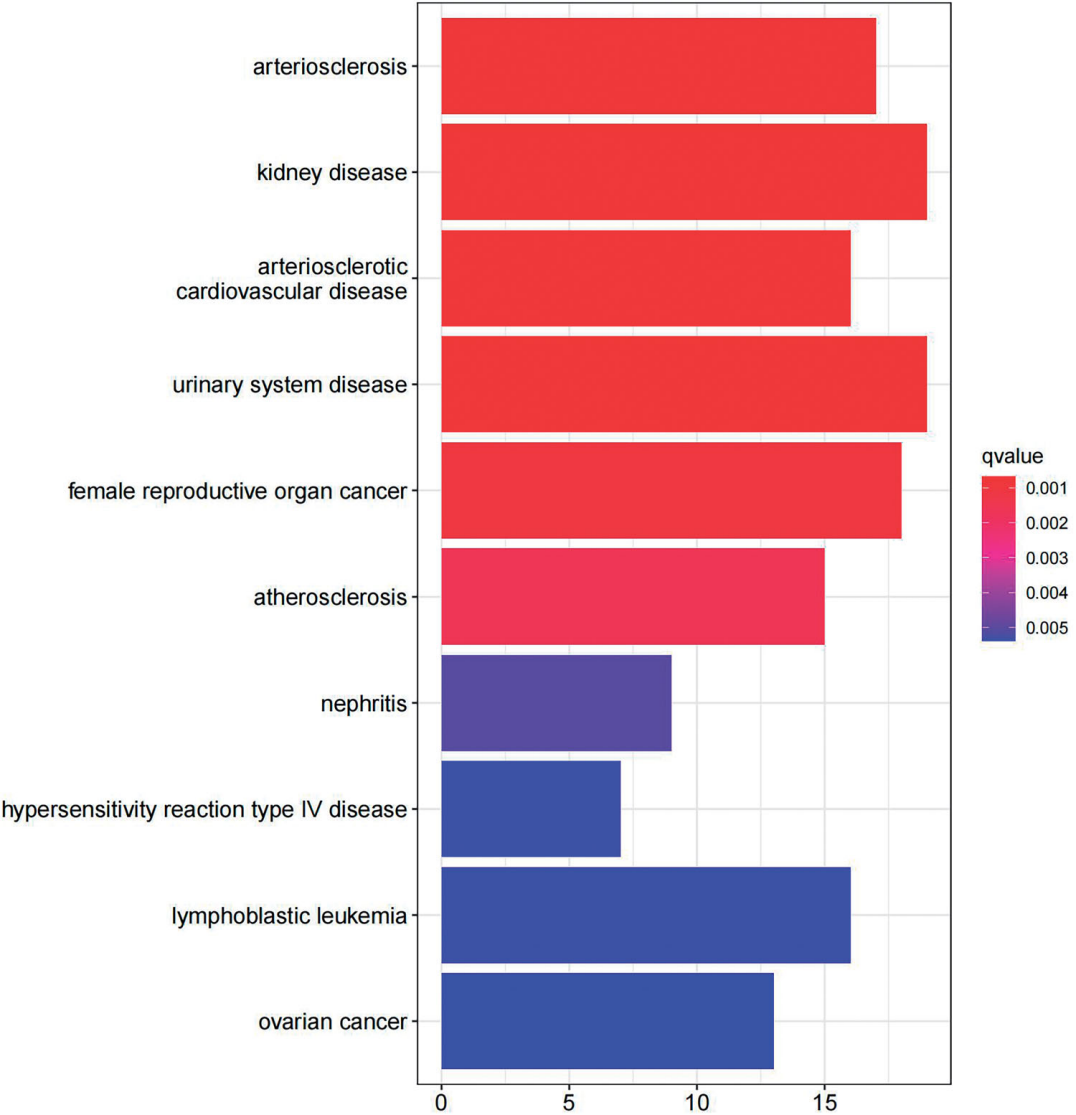
DO enrichment analysis revealed the relationship between DEGs and associated diseases. The significantly associated diseases were visualized through a barplot. The X-axis represents the number of genes enriched in each disease, and the Y-axis represents the disease name. *p* values < 0.05 indicate statistical significance. DO: disease ontology; DEGS: differentially expressed genes.

DO pathway enrichment analysis aimed to search for related diseases by investigating the function of DEGs *via* statistical R-software, and there was a significant correlation with arteriosclerosis, kidney disease and arteriosclerotic cardiovascular disease (Table 2). Meanwhile, these major enrichment diseases were visualized through a barplot (Figure 2) showing the top 10 significantly associated diseases.

**Table 2.** The related diseases of the differentially expressed genes.

Term	Genes	Count	*q* Value
DOID:2349-Arteriosclerosis	LYZ,HBA1,EGF,CD40,LGALS1,FN1,CHI3L1,KLK1,CCL5,TNC,PLTP,CTSS, UCP2,ALB,APOH,APOC1,SAA1	17	0.0007
DOID:557-Kidney disease	DUSP1,FOS,COL3A1,MT1A,CYP3A5,EGF,CD40,RGS2,TIMP1,FN1,CHI3L1, KLK1,CCL5,PTPRO,HLA-DQA1,C3,ALB,HAVCR1,LCN2	19	0.0007
DOID:2348-Arteriosclerotic cardiovascular disease	LYZ,HBA1,EGF,CD40,LGALS1,FN1,CHI3L1,CCL5,TNC,PLTP,CTSS,UCP2, ALB,APOH,APOC1,SAA1	16	0.0007
DOID:18-urinary system disease	DUSP1,FOS,COL3A1,MT1A,CYP3A5,EGF,CD40,RGS2,TIMP1,FN1,CHI3L1, KLK1,CCL5,PTPRO,HLA-DQA1,C3,ALB,HAVCR1,LCN2PTPRO,HLA-DQA1, C3,ALB,HAVCR1,LCN2	19	0.0007
DOID:120-female reproductive organ cancer	DUSP1,CCNL1,TGM2,FOS,EGF,VCAN,CD40,TIMP1,CHI3L1,KLK1,IFITM1,MMP7, HLA-DQA1,TOP2A,SLPI,CCL11,LCN2,WFDC2	18	0.001
DOID:1936-atherosclerosis	HBA1,EGF,CD40,LGALS1,FN1,CHI3L1,CCL5,TNC,PLTP,CTSS,UCP2,ALB, APOH,APOC1,SAA1	15	0.0016
DOID:10952-nephritis	DUSP1,COL3A1,MT1A,CD40,KLK1,CCL5,PTPRO,HLA-DQA1,LCN2	9	0.005
DOID:2916-hypersensitivity reaction type IV disease	COL3A1,TIMP1,CHI3L1,TNC,HLA-DQA1,HAVCR1,IGKC	7	0.0054
DOID:1037-lymphoblastic leukemia	MIR221,BGN,CYP3A5,CD40,PTPRO,CD52,MMP7,TOP2A,TLR7,CCL21,ALB, CCL11,CCL19,IGHM,CD2,FLT3	16	0.0054
DOID:2394-ovarian cancer	DUSP1,TGM2,FOS,EGF,VCAN,TIMP1,CHI3L1,KLK1,MMP7,HLA-DQA1, CCL11,LCN2,WFDC2	13	0.0054

### 4.3. DEGs gene set enrichment analysis in FSGS

**Figure 3.**
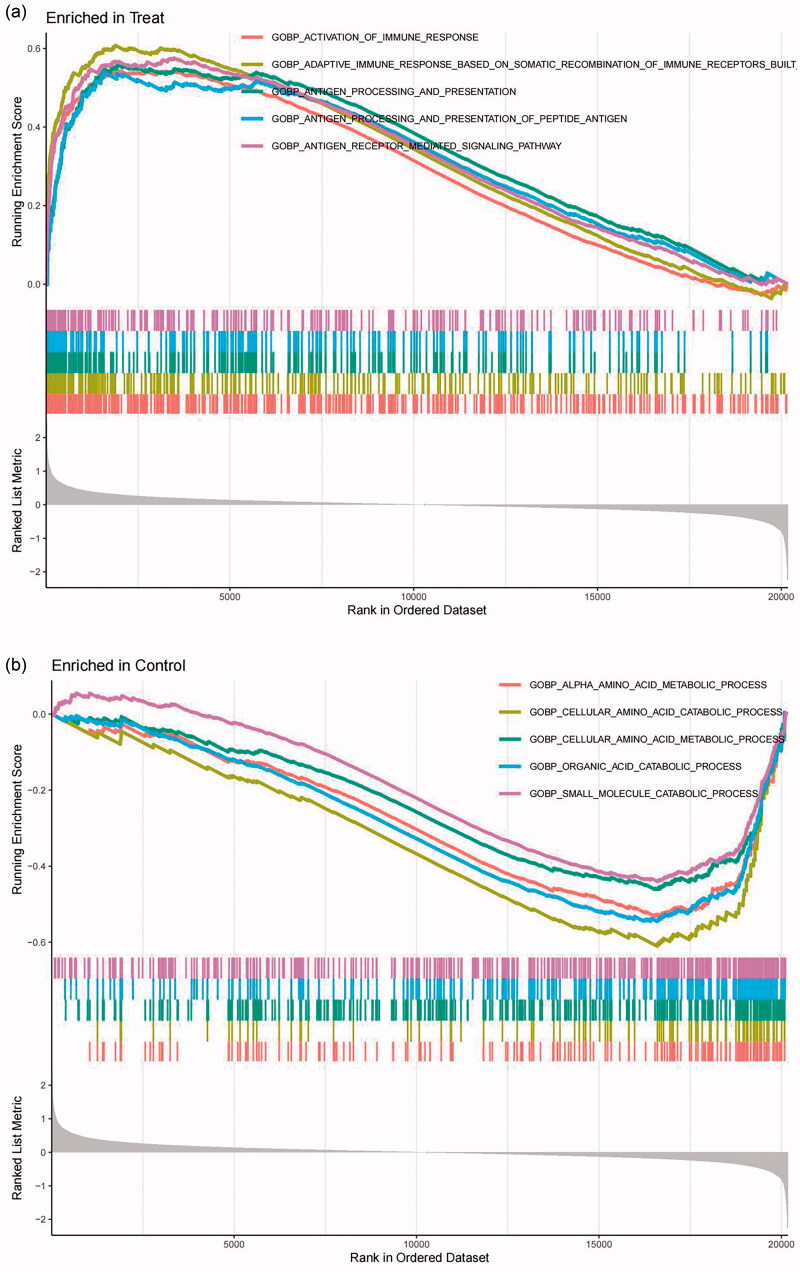
GSEA was used to perform DEG-related GO enrichment analyses. The results of GO analyses revealed the biological functional pathways significantly enriched in tubular cells from the FSGS group (a) and the control group (b), especially in BP annotation. The X-axis represents the rank in the ordered dataset, and the Y-axis represents the running enrichment score. *p* values < 0.05 indicate statistical significance. GSEA: gene set enrichment analysis; GO: gene ontology; BP: biological process.

**Figure 4.**
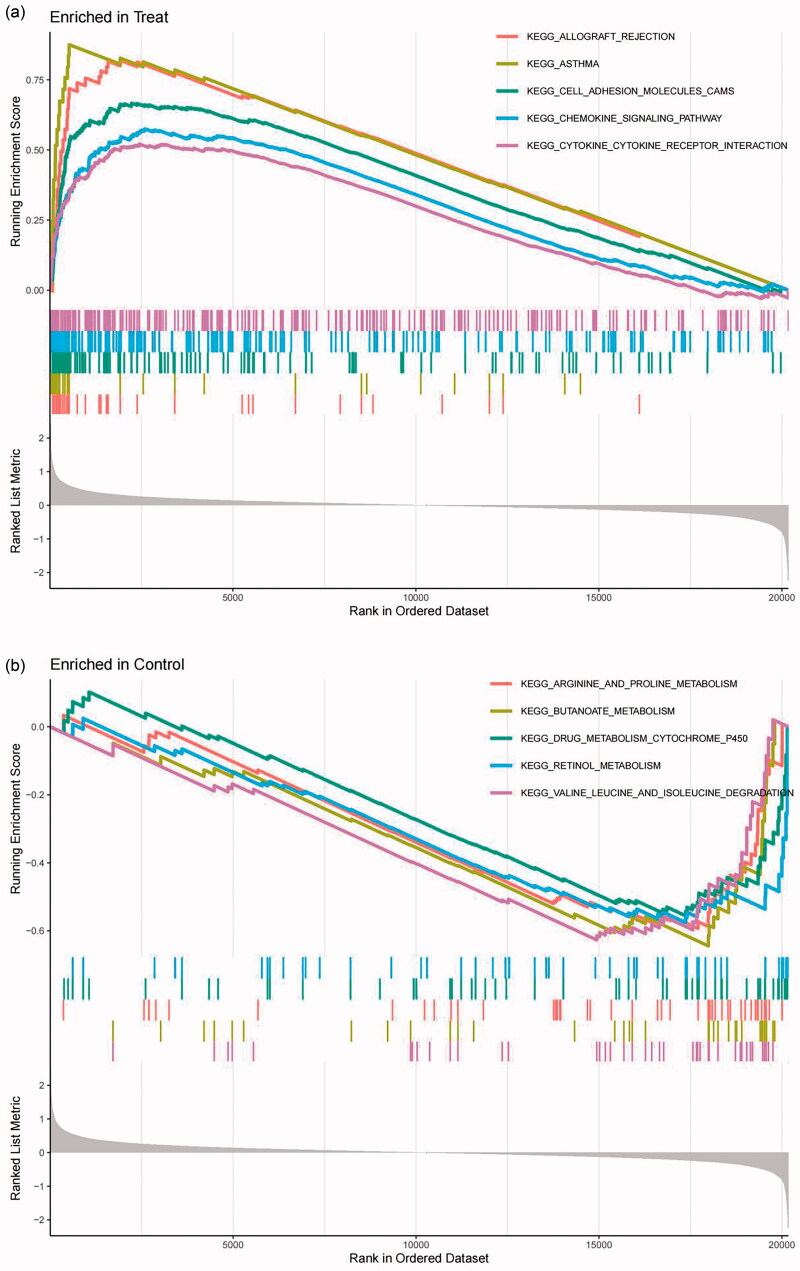
GSEA was used to perform DEG-related KEGG enrichment analyses. The results of the KEGG analyses revealed the signaling pathways significantly enriched in tubular cells from the FSGS group (a) and the control group (b). The X-axis represents the rank in the ordered dataset, and the Y-axis represents the running enrichment score. *P* values < 0.05 indicate statistical significance. GSEA: gene set enrichment analysis; KEGG: kyoto encyclopedia of genes and genomes.

GSEA was applied to perform DEG-related GO and KEGG enrichment analyses. GO enrichment analysis was carried out for three functional groups: biological processes group (BP), cellular components group (CC) and molecular functions group (MF). Specifically, the geneset ontology results were significantly enriched in BP, such as activation of immune response and adaptive immune response based on somatic recombination of immune receptors that were active in tubular cells from the FSGS group (Figure 3). KEGG signaling pathway enrichment analysis showed that allograft rejection, asthma, adhesion molecules cams, chemokine signaling pathway, and cytokine receptor interaction signaling pathways were mainly enriched in tubular cells from the FSGS group (Figure 4).

### 4.4. PPI network construction and submodule analysis

As illustrated in Table 1, the PPI network of 179 DEGs was constructed using the STRING database to study the interactions among the robust DEGs. With confidence >0.7 and after hiding the disconnected nodes, a total of 143 nodes and 108 edges were involved in the PPI network. Then, the PPI data were imported into Cytoscape software (Figure 5a). The MCC algorithm in the Cytoscape plugin cytoHubba was applied to select the top fifteen hub genes (Figure 5b). The results indicate that early growth response 1 (EGR1), fos proto-oncogene (FOS), fibronectin 1 (FN1), complement C1s (C1S), cathepsin s (CTSS), complement c2 (C2), fosB proto-oncogene (FOSB), complement c1r (C1R), cd2 molecule (CD2), transmembrane immune signaling adaptor TYROBP (TYROBP), complement c1q c chain (C1QC), complement c3 (C3), cd48 molecule (CD48), NR4A1 and DUSP1 were contributing to FSGS. GO enrichment of the hub genes is displayed in Figure 5c. The top ten elements were significantly enriched in GO categories. The BP group in the hub genes was significantly enriched in regulation of immune effector process, regulation of complement activation and regulation of humoral immune response, in addition to blood microparticles in the CC group and endopeptidase activity in the MF group.

Figure 5.PPI network construction and hub gene GO analysis. (a) The protein-protein interaction network of 179 DEGs were constructed with the STRING database. There were a total of 143 nodes, and 108 edges were involved in the PPI network. (b) The cytoHubba plugin identified the top fifteen genes as hub genes in FSGS by the MCC method. (c) The top ten elements were significantly enriched in GO categories: BP, CC and MF. The X-axis represents the gene ratio, and the Y-axis represents the GO category name. *p* values < 0.05 indicate statistical significance. PPI: protein-protein interaction; GO: gene ontology; DEGS: differentially expressed genes; STRING: a search tool for the retrieval of interacting genes; MCC: maximum correlation criteria; BP: biological processes group; CC: cellular components group; MF: molecular functions group.

**Figure:**
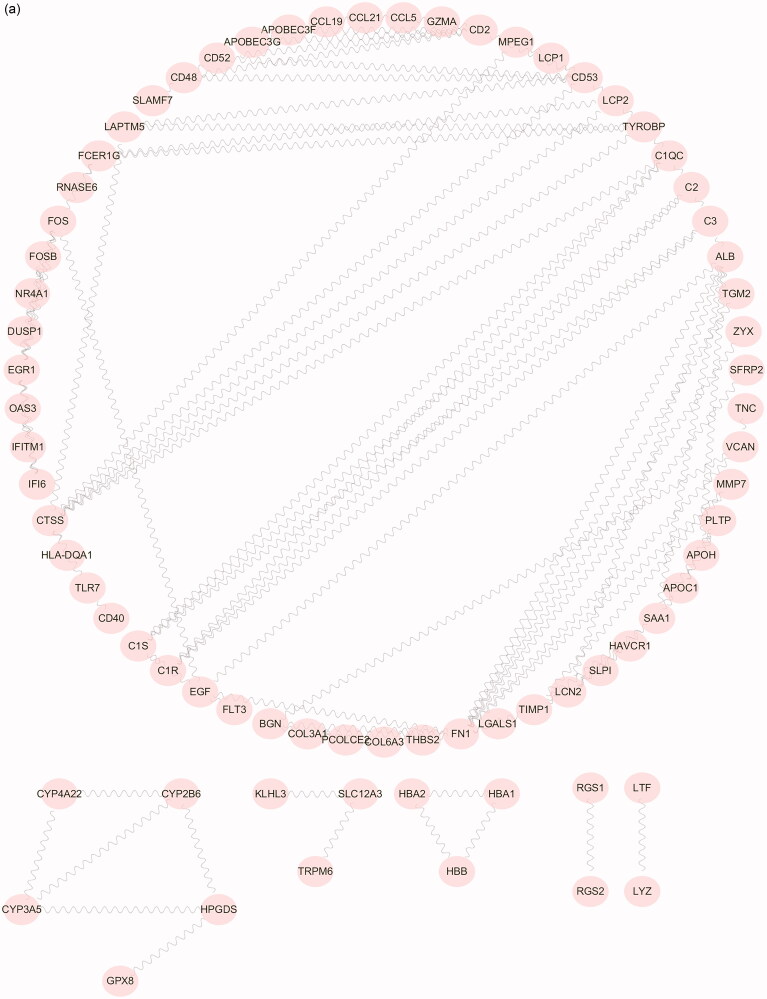

**Figure:**
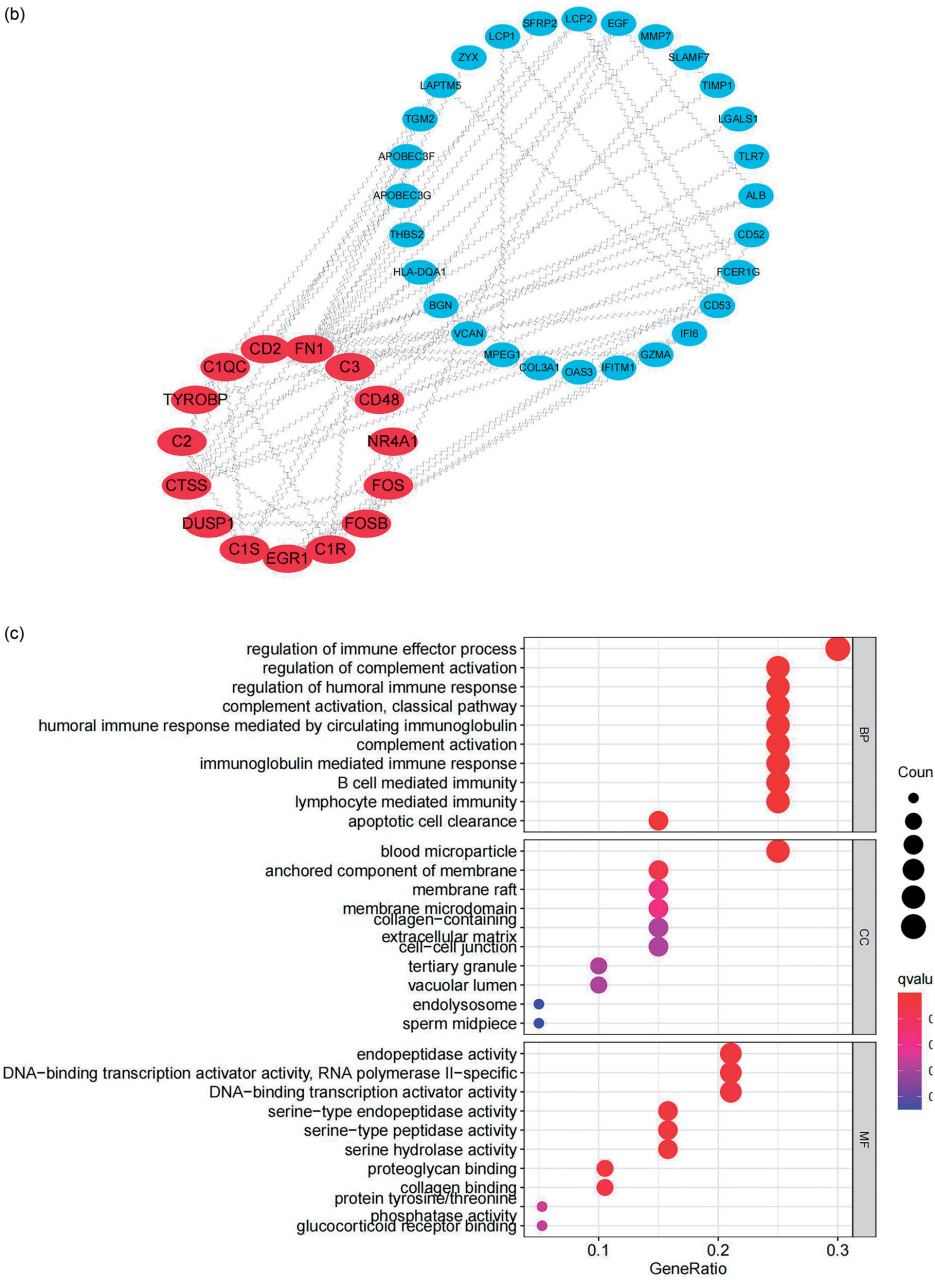

**Figure 6.**
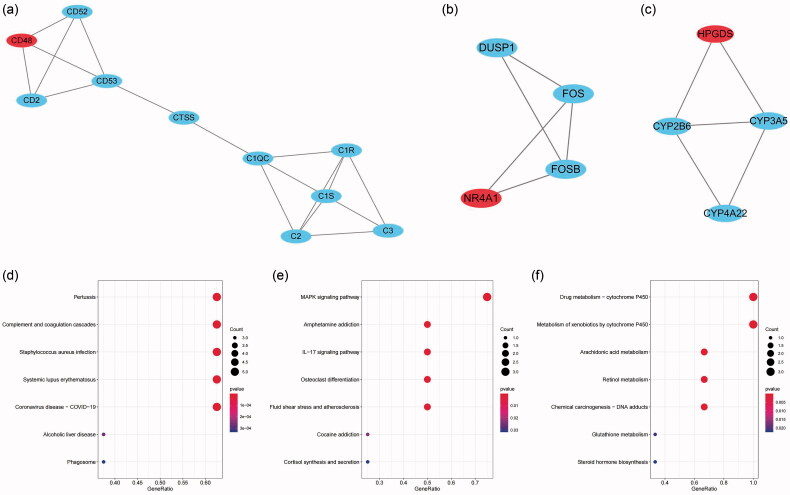
Submodule construction and KEGG pathway enrichment analysis of submodules. The three key modules screened from the PPI network using the MCODE method with a score ≥ 3 and genes ≥ 4 were identified as submodules. (a) shows module 1 with an MCODE score of 3.778, (b) shows module 2 with an MCODE score of 3.333, and (c) shows module 3 with an MCODE score of 3.333. (d–f) show the top 7 functional pathways associated with the genes in modules 1–3 through KEGG pathway enrichment analysis. The X-axis represents the gene ratio, and the Y-axis represents the significantly enriched KEGG pathways of the module. *p* values < 0.05 indicate statistical significance. KEGG: kyoto encyclopedia of genes and genomes; PPI: protein-protein interaction; MCODE: molecular complex detection.

In addition, three key modules with a score ≥ 3 and genes ≥ 4 were screened from the whole network by MCODE (Figure 6a–c). In the robust DEGs in module 1 with a score of 3.778, CD48, CD52, CD53, C1QC, CD2, C3, CTSS, C1R, C1S and C2 were hub nodes; in module 2 with a score of 3.333, DUSP1, NR4A1, FOS and FOSB were hub nodes; and in module 3 with a score of 3.333, hematopoietic prostaglandin D synthase (HPGDS), cytochrome P450 family 2 subfamily B member 6 (CYP2B6), cytochrome P450 family 3 subfamily A member 5 (CYP3A5) and cytochrome P450 family 4 subfamily A member 22 (CYP4A22) were hub nodes. The enrichment pathways of the three modules are displayed in Figure 6d–e. The pathways in module 1 were mainly enriched in pertussis, complement and coagulation cascades, and the staphylococcus aureus infection pathway; those in module 2 were mainly enriched in the MAPK signaling pathway, amphetamine addiction and the IL − 17 signaling pathway; and those in module 3 were mainly enriched in drug metabolism-cytochrome P450, metabolism of xenobiotics by cytochrome P450 and the arachidonic acid metabolism pathway.

### 4.5. Construction of a LASSO model and SVM-RFE for candidate gene biomarkers in FSGS

**Figure 7.**
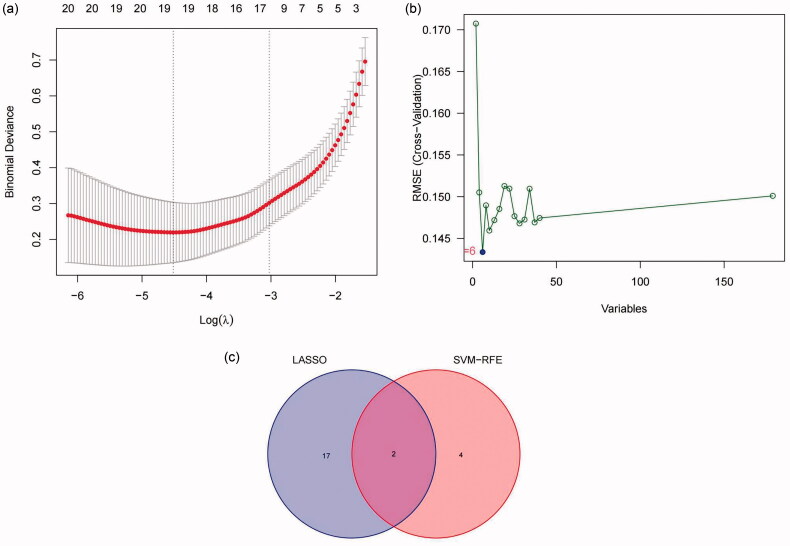
LASSO model and SVM-RFE were used to mine the gene biomarkers of FSGS. (a) Nineteen genes were extracted as gene biomarkers of FSGS *via* LASSO regression. (b) Six genes were extracted as gene biomarkers of FSGS using the SVM-RFE algorithm. (c) A total of 2 overlapping genes between LASSO regression and the SVM-RFE algorithm were identified. LASSO: least absolute shrinkage and selection operator; SVM-RFE: support vector machine recursive feature elimination.

**Figure 8.**
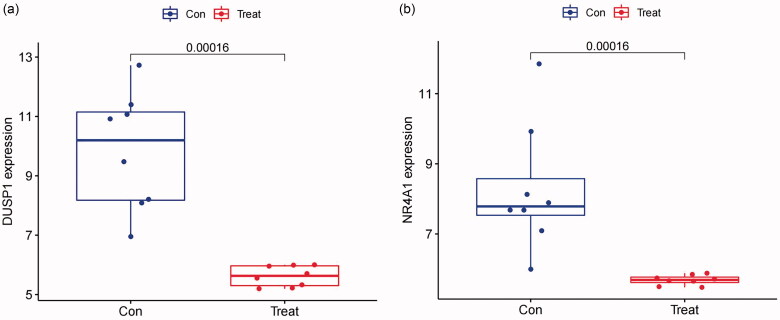
The present study utilized the GSE125779 Series Matrix File to validate the levels of expression of the two characteristics, renal tubular samples of the control group and renal tubular samples of the FSGS group. (a) DUSP1 (b) NR4A1. The X-axis represents the sample grouping, and the Y-axis represents the target gene expression. *p* values < 0.05 indicate statistical significance. DUSP1: dual specificity phosphatase 1; NR4A1: nuclear receptor subfamily 4 group A member 1.

The present study utilized two machine learning algorithms, the LASSO model and SVM-RFE, to mine the gene biomarkers associated with FSGS from DEGS. By LASSO regression, we extracted nineteen genes, which narrowed the range of DEGs (Figure 7a). While using the SVM-RFE algorithm, we mined six characteristic genes in FSGS (Figure 7b). Meanwhile, two duplicate genes between the machine learning algorithms were identified, namely, DUSP1 and NR4A1, which were also selected as hub genes by the MCC algorithm in cytoHubba, as well as hub nodes involved in the submodule by the MCODE algorithm (Figure 7c). To validate the two identified genes, the diagnostic model was established by a logistic regression algorithm (Figure 8). The GSE125779 Series Matrix File was utilized to validate the expression levels of DUSP1 and NR4A1, which were significantly lower in FSGS tubular samples than in control samples.

### 4.6. Value of gene biomarkers in FSGS

**Figure 9.**
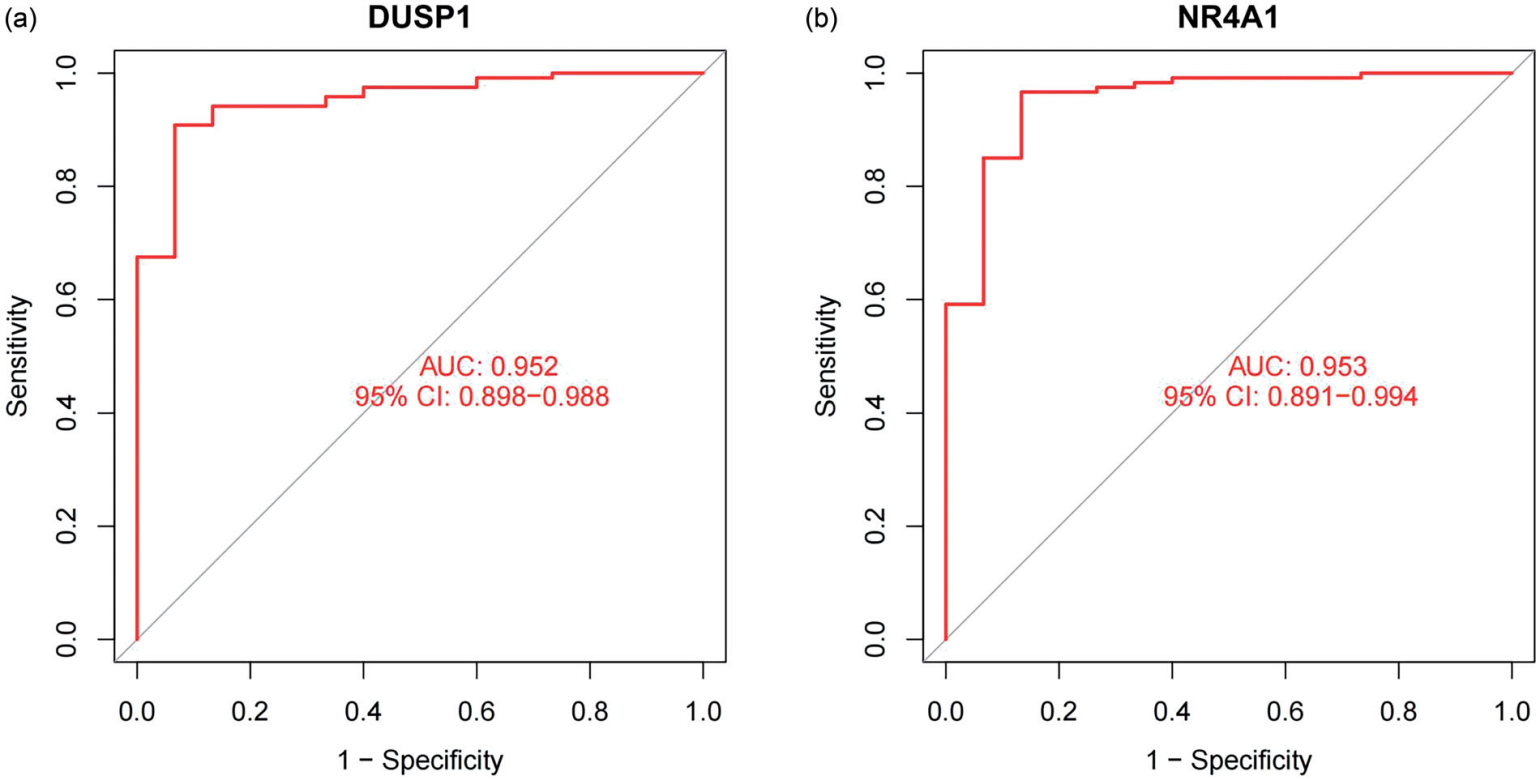
ROC curve analyses were used to investigate the logistic regression model. (a) DUSP1 (b) NR4A1. The X-axis represents the (1-specificity), and the Y-axis represents the sensitivity. *P* values < 0.05 indicate statistical significance. ROC: Receiver operating characteristic; DUSP1: dual specificity phosphatase 1; NR4A1: nuclear receptor subfamily 4 group A member 1.

**Figure 10.**
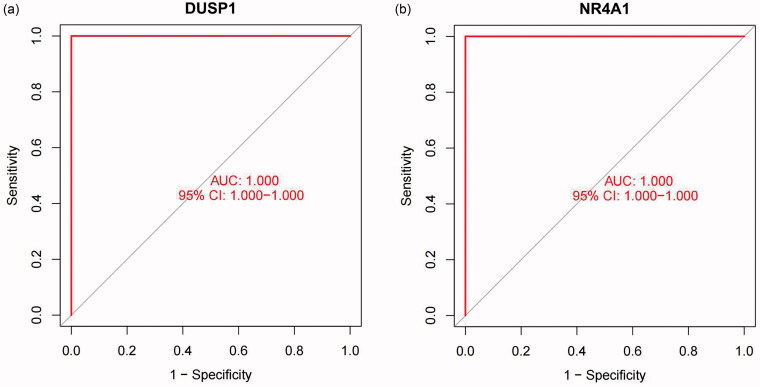
The GSE125779 Series Matrix File was used to validate the logistic regression model *via* ROC. (a) DUSP1 (b) NR4A1. The X-axis represents the (1-specificity), and the Y-axis represents the sensitivity. *P* values < 0.05 indicate statistical significance. ROC: Receiver operating characteristic; DUSP1: dual specificity phosphatase 1; NR4A1: nuclear receptor subfamily 4 group A member 1.

The present study also utilized ROC curve analyses to investigate the logistic regression model. Based on the previous calculations, the candidate gene markers of the FSGS renal tubules are useful for diagnosing FSGS. The AUC was 0.952 (95% CI: 0.898–0.988) for DUSP1 and 0.953 (95% CI: 0.891–0.994) for NR4A1 (Figure 9). Meanwhile, we validated the logistic regression model with the external dataset GSE125779, and the results demonstrated powerful predictive capabilities. The AUC was 1.000 (95% CI: 1.000–1.000) for DUSP1 and 1.000 (95% CI: 1.000–1.000) for NR4A1 (Figure 10).

### 4.7. Analysis of immune cell infiltration in FSGS

The immune infiltration in FSGS was calculated *via* the CIBERSORT algorithm. We contrasted the immune cell components between the FSGS samples and control samples. The results indicated that activated mast cells (*p* < 0.001) and naive CD4 T cells (*p* = 0.0019) in the FSGS group were remarkably lower than those in the control group, while gamma delta T cells (*p* = 0.0026) in the FSGS group were remarkably higher than those in the control group (Figure 11a,b). The interaction between immune cells is visualized in Figure 11c. The results demonstrated that activated mast cells had a significant negative correlation with resting mast cells (*r* = −0.22), while they had a significant positive correlation with M1 macrophages (*r* = 0.25). CD4 naive T cells had a significant negative correlation with CD8 T cells (*r* = −0.19) but a significant positive correlation with M0 macrophages (*r* = 0.26). Gamma delta T cells had a significant negative correlation with monocytes (*r* = −0.38) but a significant positive correlation with M1 macrophages (*r* = 0.24).

Figure 11.Correlation analysis of infiltrating immune cells. (a) The contrast of immune cell components between the control group and the FSGS group. The X-axis represents the immune cells, and the Y-axis represents the fraction. (b) The differences in immune cell infiltration between the control group and the FSGS group. The X-axis represents the sample type, and the Y-axis represents the relative percent. (c) The correlation of the infiltration of innate immune cells. The X-axis and the Y-axis represent the classification of immune cells. *p* values < 0.05 indicate statistical significance.

**Figure:**
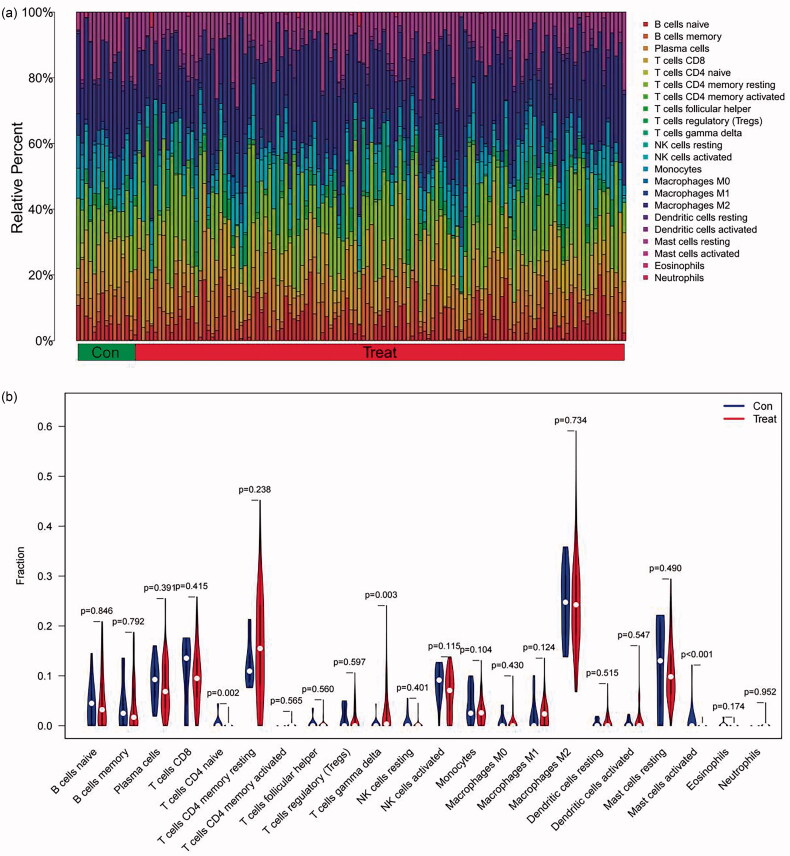

**Figure:**
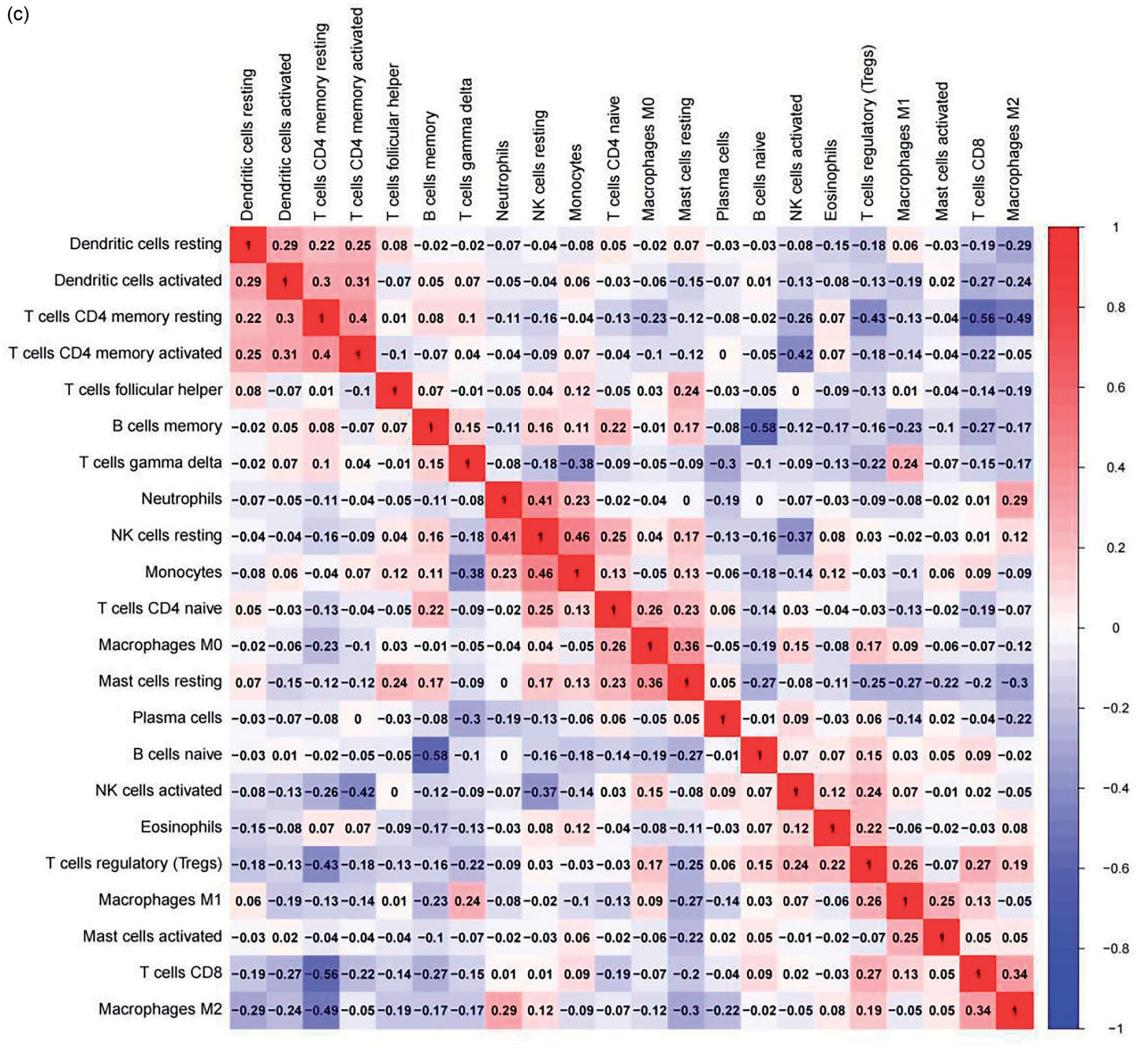

### 4.8. Correlation Analysis between the identified genes and immune cell infiltration

**Figure 12.**
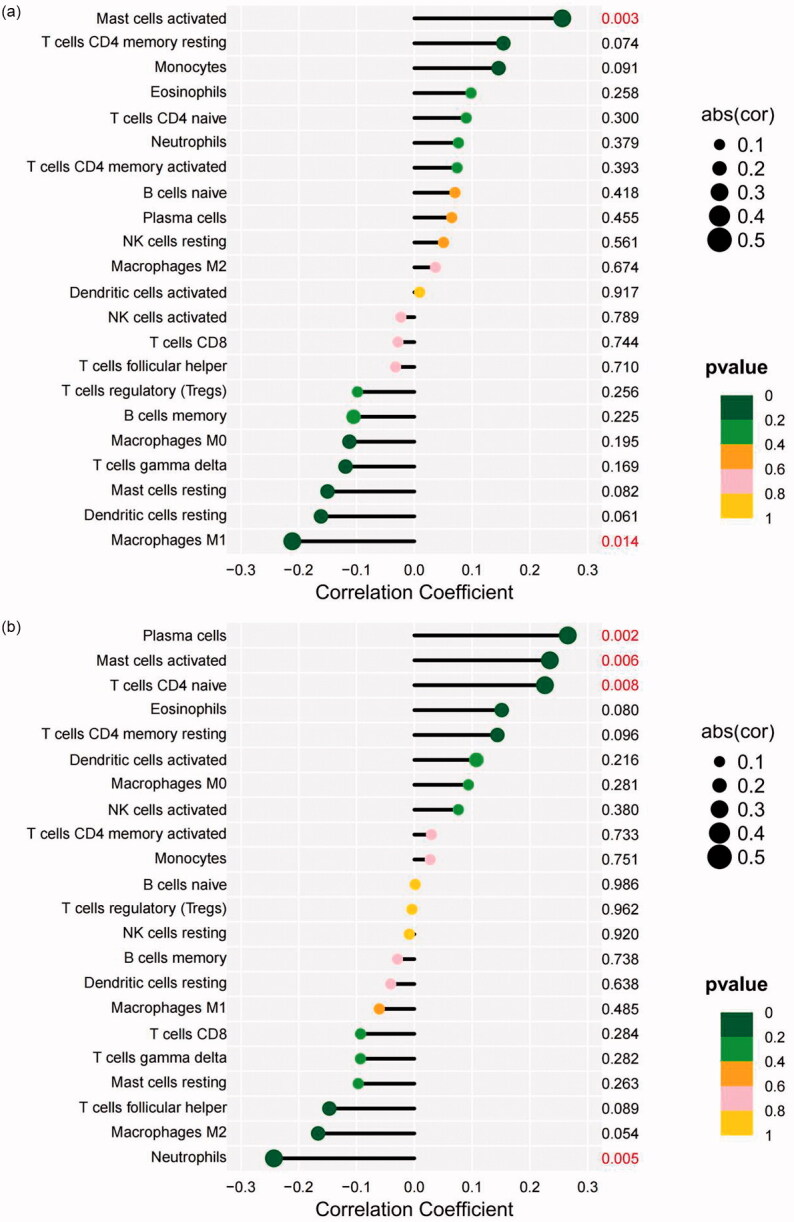
The relationship between the two selected genes and infiltrating immune cells. (a) DUSP1. (b) NR4A1. The X-axis represents the correlation coefficient, and the Y-axis represents the immune cells. *p* values < 0.05 indicate statistical significance. DUSP1: dual specificity phosphatase 1; NR4A1: nuclear receptor subfamily 4 group A member 1.

Pearson correlation analysis was applied to examine the relationship between the two selected genes and infiltrating immune cells. The results indicated that DUSP1 had a significant negative correlation with M1 macrophages (*r* = −0.211, *p* = 0.014), while it had a significant positive correlation with activated mast cells (*r* = 0.256, *p* = 0.003) (Figure 12a). NR4A1 had a significant negative correlation with neutrophils (*r* = −0.243, *p* = 0.005) but had a significant positive correlation with plasma cells (*r* = 0.266, *p* = 0.002), activated mast cells (*r* = 0.235, *p* = 0.006), and naive CD4 T cells (*r* = 0.226, *p* = 0.008) (Figure 12b).

## 5. Discussion

FSGS is a syndrome with a severe economic burden, a low cure rate and many complications, and its occurrence and development are closely related to the immune response. The tubule injury is closely correlated with progressive loss of kidney function. The progression of segmental sclerosis in a single nephron to global sclerosis, and the progression from glomerular lesions to focal tubular atrophy and renal interstitial fibrosis are important links in the progression and worsening of FSGS to ESRD, this is why patients with FSGS often have severe tubulointerstitial pathology.

The activation of the intrarenal complement system is involved in the progression of renal disease, and the proximal tubule is a central target of the activated complement cascade and is the site where abnormally filtered plasma proteins and complement factors bind and promote injury [37]. In the present study, three gene chips, GSE108112, GSE133288 and GSE121211, were downloaded from GEO datasets, and a follow-up comprehensive bioinformatics analysis was conducted. The GSEA results showed that the immune processes and immune pathways were mostly associated with FSGS. Based on the functional analysis of the hub genes and hub nodes involved in the submodule, the results strongly suggest that the immune response is also closely related to the occurrence and development of FSGS.

The complement system is a proteolytic cascade in blood plasma and a mediator of innate immunity, one of the main consequences of complement activation is the recruitment of inflammatory and immunocompetent cells. Inflammatory immune responses require leukocyte recruitment to inflammatory sites by exogenous inflammation. In order to carry out the important immune functions in the inflammatory site, the blood circulation of T lymphocytes must be arrested, adhered, migrated and transmigrated on the endothelial surface and coordinate the progress of steps is coordinated by cellular adhesion molecules, chemokines and selectins presented on the endothelium [38]. These are consistent with the functional enrichment confirmed in this study.

Proteinuria is an indicator of the prognosis of progressive nephropathy. Abnormally filtered bioactive macromolecules interact with PTECs, leading to the development of proteinuric nephropathy [39]. Previous studies have shown that albumin may stimulate proximal tubular cells to secrete chemokines; if activated, normal T cells are expressed and secreted, and the macrophage migration inhibitory factor enters the basal lateral culture medium. The polarized secretion of these chemoattractants *in vivo* is intended to promote the recruitment of monocytes and lymphocytes into the renal intersection [40]. The mitogen-activated protein kinase (MAPK) cascade is involved in the cellular functions of cell migration, and it is well established that activation of the extracellular signal-regulated kinase (ERK) and p38 MAPK signal transduction pathways play an important role in the inflammatory response [40]. Meanwhile, the p38 MAPK pathway is involved in TGF-β1-induced epithelial-mesenchymal transition in renal tubular epithelial cells [41], which may be implicated in chronic kidney disease associated with proteinuria and progressive tubulointerstitial injury.

Two specific genes were identified by the LASSO model and SVM-RFE, DUSP1 and NR4A1, and they were validated by an external dataset. DUSP1 is also called MKP1, and the protein encoded by this gene can dephosphorylate MAPK1/ERK2. DUSP1 has a decisive effect on the inflammatory reaction, appears to be a central mediator for resolving inflammation, and overexpression of DUSP1 has been proposed as a significant mechanism involved in GC actions [42]. Sheng J et al. showed that DUSP1 reduced mitochondrial damage caused by hyperglycemia, while a decrease in DUSP1 expression was related to glucose metabolism disorders, renal dysfunction, renal fibrosis and glomerular apoptosis [43]. Lu C et al. also showed that DUSP1 was decreased in HK-2 cells under hyperglycemic conditions, but in HG-treated HK-2 cells, overexpression of DUSP1 fractionally regenerated the autophagic flux and optimized the mitochondrial function. Meanwhile, by increasing parkin expression, the production of reactive oxygen species and cell apoptosis were decreased [44].

NR4A1, also called NUR77, is a regulator of tissue responses and is associated with fundamental cellular processes involving inflammation, proliferation, differentiation, and survival [45], especially leukocyte infiltration and the release of cytokines in response to injury. NR4A1 has been identified as an endogenous inhibitor of the conversion of TGF-β signaling, which is a promising target for the recovery of mesenchymal homeostasis and the treatment of fibrosis [46]. Zeng X et al. showed that the loss of NR4A1 stimulated fibrogenesis in mice with endometriosis by increasing TGF-β-dependent elevated expression [47]. NR4A1 is expressed in mouse kidney cells and cultured renal cell lines [48]. Westbrook L et al. indicated that the severity of tubular atrophy, tubular casts, and interstitial fibrosis increased observably in NR4A1-deficient mice and was coupled with a significant increase in immune cell infiltration, mainly macrophages and, to a lesser extent, T cells and B cells, thereby increasing kidney damage and renal dysfunction [49]. Wang S et al. showed that JMJD1A/NR4A1 signaling could regulate the progression of renal tubular epithelial interstitial fibrosis in HK-2 cells [50], and maintenance of NR4A1 may be an effective strategy for blocking renal tubulointerstitial fibrosis and improving renal function in elderly individuals [51].

Pharmacological and genetic studies indicate that immune cell infiltration into the kidney amplifies the disease process [52], so it is significant to calculate immune infiltration by the CIBERSORT algorithm to find multiple immune subtypes which were closely related to crucial FSGS biological processes. Increased infiltration of gamma delta T cells and decreased infiltration of activated mast cells and naive CD4 T cells may be related to the development of FSGS through tubular injury and tubulointerstitial inflammation.

In the correlation analysis, the gene biomarkers DUSP1 and NR4A1 were both significantly correlated with infiltrating immune cells and activated mast cells. Mast cells (MCs) regulate inflammatory reactions as well as tissue repair in human diseases and they increase considerably in various renal diseases. Activation of MCs regulates innate immunity and adaptive effector responses, and several chemokines, cytokines, and proteases released by MCs have been independently observed in various kidney diseases. The MC-specific protease tryptase is able to activate the GPCR protease PAR-2, which is widely expressed in the kidney, especially in tubular epithelial cells, and its activation triggers strong inflammatory and fibrotic reactions [53].

In contrast, there are contradictory reports on the role of MCs in the pathogenesis of various renal diseases. Miyazawa S et al. demonstrated that MCs play a protective role in interstitial fibrosis with puromycin aminonucleoside nephrosis by inhibiting heparin's production of TGF-β [54]. Kim DH et al. found that MC-deficient mice had higher levels of renal tubular injury and more interstitial fibrosis [55]. It was also demonstrated that DUSP1 could inhibit the phosphorylation of MAPK in MCs [56], while NR4A1 can function as a proinflammatory mediator in activated immune cells that regulate mucosal MC activation [57], the above are the possible mechanisms how DUSP1 and NR4A1 activate MC.

Consistent with the previous evidence, our study results show that activated infiltrating immune cells, especially MCs, play a crucial role in FSGS, strongly indicating that the immune response is an important factor in its pathology, which should be the focus of future research.

Due to the limited number of samples in this retrospective study, the functions of the two identified genes and immune cell infiltration in FSGS were deduced by bioinformatics analysis; hence, further *in vitro* and *in vivo* experiments are required to validate novel biomarkers in the future.

## 6. Conclusion

In this study, DUSP1 and NR4A1 were identified as sensitive potential renal tubular biomarkers in FSGS diagnosis. Activated MCs have a significant effect on the occurrence and development of FSGS and are expected to become therapeutic targets.

## Data Availability

Publicly available datasets were analyzed in this study. All raw data are available in GEO datasets (GSE108112, GSE133288, GSE121211 and GSE125779).
